# Neuroprotective effect of ACTH on collagenase-induced peri-intraventricular hemorrhage in newborn male rats

**DOI:** 10.1038/s41598-020-74712-7

**Published:** 2020-10-20

**Authors:** Camila A. Martins, Laura Tartari Neves, Marina M. B. P. de Oliveira, Pamela Brambilla Bagatini, Rafaela Barboza, Régis Gemerasca Mestriner, Léder Leal Xavier, Alberto A. Rasia-Filho

**Affiliations:** 1 Programa de Pós-Graduação em Biociências, Universidade Federal de Ciências da Saúde de Porto Alegre (UFCSPA), Porto Alegre, RS 90170-050 Brazil; 2 Departamento de Ciências Básicas da Saúde/Fisiologia, Universidade Federal de Ciências da Saúde de Porto Alegre, R. Sarmento Leite 245, Porto Alegre, RS 90170-050 Brazil; 3 Laboratório de Biologia Celular e Tecidual, Escola de Ciências, Pontifícia Universidade Católica do Rio Grande do Sul, PUCRS, Porto Alegre, 90619-900 Brazil; 4 Programa de Pós-Graduação em Neurociências, Universidade Federal do Rio Grande do Sul, Porto Alegre, RS 90170-050 Brazil

**Keywords:** Diseases of the nervous system, Glial biology

## Abstract

Peri-intraventricular hemorrhage (PIVH) is a common and serious prematurity-related complication in neonates. Adrenocorticotropic hormone (ACTH) has neuroprotective actions and is a candidate to ameliorate brain damage following PIVH. Here, we tested the efficacy of ACTH_1-24_ on a collagenase-induced lesion of the germinal matrix (GM) in newborn male rats. Animals received microinjection of the vehicle (PBS, 2 µl) or collagenase type VII (0.3 IU) into the GM/periventricular tissue on postnatal day (PN) 2. Twelve hours later pups received microinjection of either the agonist ACTH_1-24_ (0.048 mg/kg), or the antagonist SHU9119 (antagonist of MCR3/MCR4 receptors, 0.01 mg/kg), or their combination. Morphological outcomes included striatal injury extension, neuronal and glial cells counting, and immunohistochemical expression of brain lesion biomarkers ipsilateral and contralateral to the hemorrhagic site. Data were evaluated on PN 8. Collagenase induced PIVH and severe ipsilateral striatal lesion. ACTH_1-24_ dampened the deleterious effects of collagenase-induced hemorrhage in significantly reducing the extension of the damaged area, the striatal neuronal and glial losses, and the immunoreactive expression of the GFAP, S100β, and NG2-glia biomarkers in the affected periventricular area. SHU9119 blocked the glial density rescuing effect of ACTH_1-24_. ACTH_1-24_ could be further evaluated to determine its suitability for preclinical models of PVH in premature infants.

## Introduction

Approximately 15 million premature children are born each year, representing 5–18% of all births in the world^1,2^. Complications due to prematurity are the leading cause of death among children under 5 years of age, accounting for 1 million deaths^2^. Most survivors show long-term neuropsychological sequelae and severe developmental impairments^3,4^. Peri-intraventricular hemorrhage (PIVH) is one of the most serious prematurity-related illnesses^5,6^ because of its incidence (15–30% of infants born at less than 32 weeks’ gestational age) and the severity of brain injury^7,8^. The incidence of PIVH is inversely proportional to the gestational age and the newborn weight^3,9,10^, with a higher risk in male newborns^11^.

In premature infants, PIVH is usually unilateral, asymmetric and initiates in the germinal matrix (GM)^5,6^, a highly vascularized area located near the head of the caudate nucleus and below the ventricular ependymal^7^. The GM is responsible for active neuronal and glial precursor cell proliferation and migration during brain maturation^6^. The pathogenesis of PIVH is multifactorial and related to vascular, intravascular, and extravascular factors^7,8^. They involve the fragile capillary wall of the subependymal GM in the subventricular zone of the brain^7^, deficient/immature extravascular support, disturbances in cerebral blood pressure and flow pattern^5,7,8,10^, and impaired coagulation in some cases^7,8^. As a consequence of PIVH, medullary venous flow obstruction, thrombi and infarction can lead to perivascular hemorrhage and periventricular hemorrhagic necrosis^7,8^. Furthermore, the GM is particularly vulnerable to insults due to the thinness of the blood vessel walls and immature structure of the capillary bed, paucity of pericytes, immaturity of the basal lamina and fragility of the blood brain barrier^5–7^.

Regarding the time of PIVH onset, 50% of infants are affected on the post-natal day (PN) one, with additional 25% and 15% on PN 2 and 3, respectively^7^, based on clinical suspicion and defined by serial cranial ultrasonography^7,8^. Brain lesion progression occurs in up to 40% of the affected infants, usually reaching its maximal extent within 3–5 days of the initial diagnosis^7,9^. The brain damage caused by vascular rupture in the GM varies from isolated subependymal hemorrhage, adjacent intraventricular hemorrhage without ventricle enlargement or with such enlargement to bleeding extending into the brain parenchyma beyond the ganglionic eminence, graded I–IV respectively^5^, see relevant interpretation in^7,8^. Three basic clinical syndromes accompany PIVH, usually as a clinically silent syndrome, a saltatory deterioration or, less often, a catastrophic deterioration^7^. Clinical presentation varies depending on the extension of PIVH and brain damage over time^8^. PIVH can lead to post-hemorrhagic hydrocephalus, periventricular leukomalacia, gray and white matter dysmaturation, cerebellar underdevelopment, cerebral palsy, seizures, and cognitive deficits^6–8,10^.

The microinjection of collagenase in the GM is a useful animal model of PIVH in neonatal rats^12,13^. Collagenase catalyzes the hydrolysis of collagen in the basal lamina of blood vessels, dissolves the extracellular matrix surrounding capillaries and induces bleeding^12^. The collagenase-induced PIVH and the resulting brain damage cause high-grade lesions with long-term motor and cognitive deficits in rats^12^. Indeed, ventricular dilation and tissue injury in the periventricular region near the intraventricular extending hematoma are common findings in this PIVH rat model^5,13^. Moreover, the overexpression of some neuroglial proteins in the neonatal brain can be used as biochemical markers to establish the severity of this nervous tissue injury^4,14,15^. For example, increased levels or expression of glial fibrillary acid protein (GFAP), S100 calcium-binding protein β (S100β), and neural/glial antigen 2 (NG2), a chondroitin sulfate proteoglycan, are directly related to the extension of brain damage and cellular death^4,14,16^. GFAP is a cytoskeletal intermediate filament mostly expressed in mature astrocytes^17^. S100β is a calcium-binding protein physiologically expressed and released mostly from glia cells^4,14^. Among other cells, NG2 expression labels oligodendrocyte precursor cells, the largest proliferative progenitor population cell in the nervous tissue, broadly named NG2-glia^16,18^.

Adrenocorticotropic hormone (ACTH) has neuroprotective effects in hemorrhagic shock by activating anti-inflammatory pathways in rats^19^. ACTH can also protect neurons and oligodendroglia from apoptosis, excitotoxicity, oxidative stress, and inflammatory-related damage^20–22^. ACTH and melanocyte-stimulating hormones, collectively named melanocortins, act on receptors (MCRs) numbered from MCR1 to MCR5^18^. MCR1, MCR3, and MCR4 exist within the central nervous system, and MCR4 modulates energy homeostasis, inflammation, neuroprotection, and long-lasting functional recovery after brain injury^20,23^.

We studied the neuroprotective effects of ACTH on morphological parameters and on the expression of biomarkers in a rat model of PIVH. The microinjection of collagenase in the newborn rat GM mimics some aspects of PIVH injury in the early critical period of life in children. The present study aimed to: (1) employ the unilateral collagenase-induced bleeding in the GM, as a PIVH model in newborn male rats, one day after birth; (2) determine the density of neurons and glia cells in the ipsilateral microinjected site; (3) assess GFAP, S100β, and NG2 immunoreactivity in the lesioned brain parenchyma using immunohistochemical approach; (4) test the neuroprotective effects of microinjected ACTH following collagenase-induced PIVH on the same above-mentioned outcomes; and, (5) determine whether the effects of ACTH could be counteracted by the concomitant use of an antagonist of MCR3 and MCR4.

## Results

**Figure 1:**
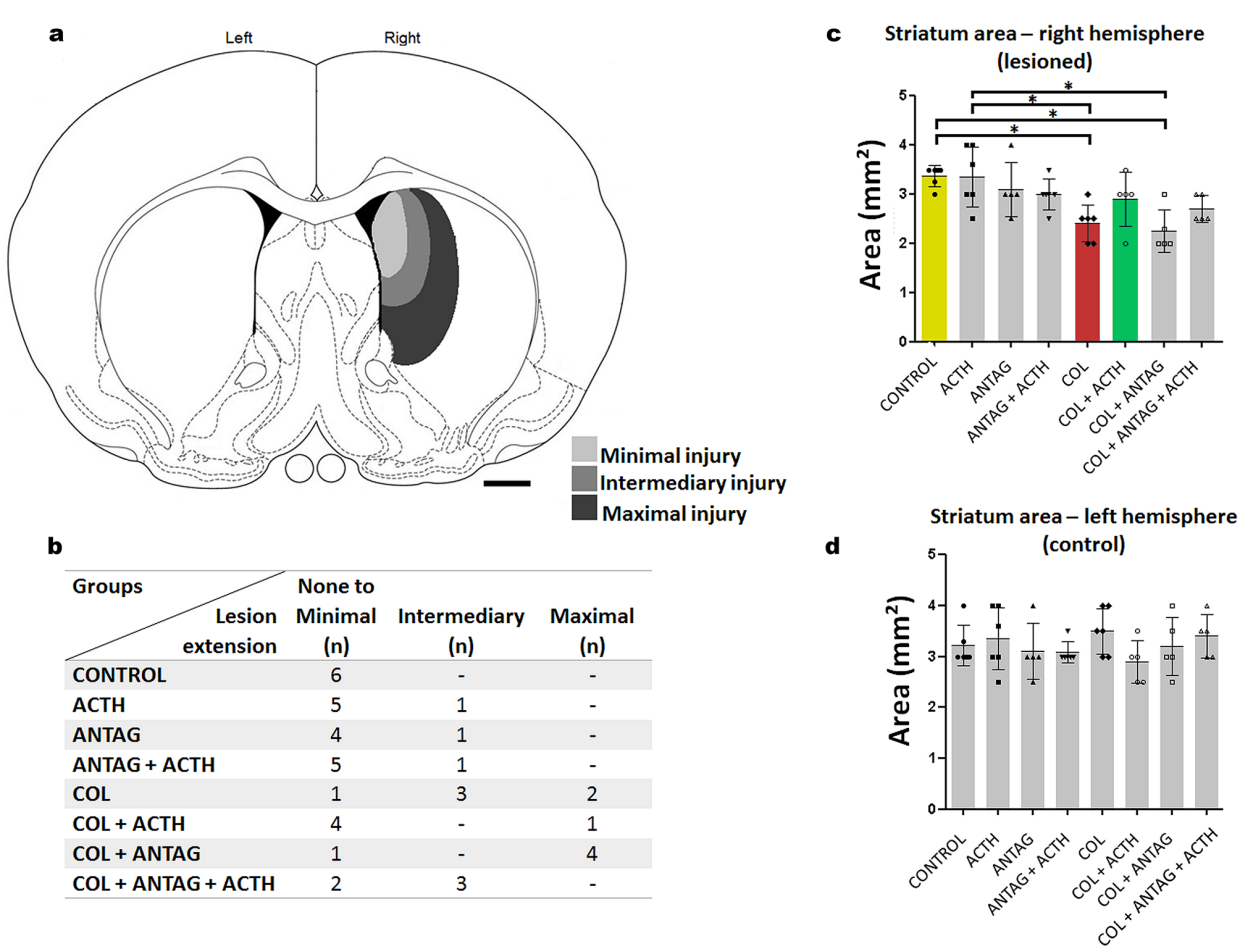
(**a**) Representative illustration of minimal, intermediary, and maximal injury area in the striatum after microinjections directed to the germinal matrix of the right hemisphere. Adapted from^53^. Scale bar = 1 mm. (**b**) Number of newborn male rats in each experimental group showing injury in the ipsilateral striatum. Note more extensive lesions in groups microinjected with collagenase. (**c**) Values (mean ± standard deviation in mm^2^) for the area of the right striatum in the experimental groups (n = 5 in ANTAG, COL + ACTH, COL + ANTAG, and COL + ACTH + ANTAG groups; n = 6 in Control, ACTH, ACTH + ANTAG, and COL ones). Symbols represent individual values. *p < 0.05 for each comparison indicated. Note the control, collagenase and collagenase plus ACTH data. (**d**) Corresponding values (mean ± standard deviation in mm^2^) for the contralateral, left striatum. No statistically significant difference was found between groups.

**Figure 2:**
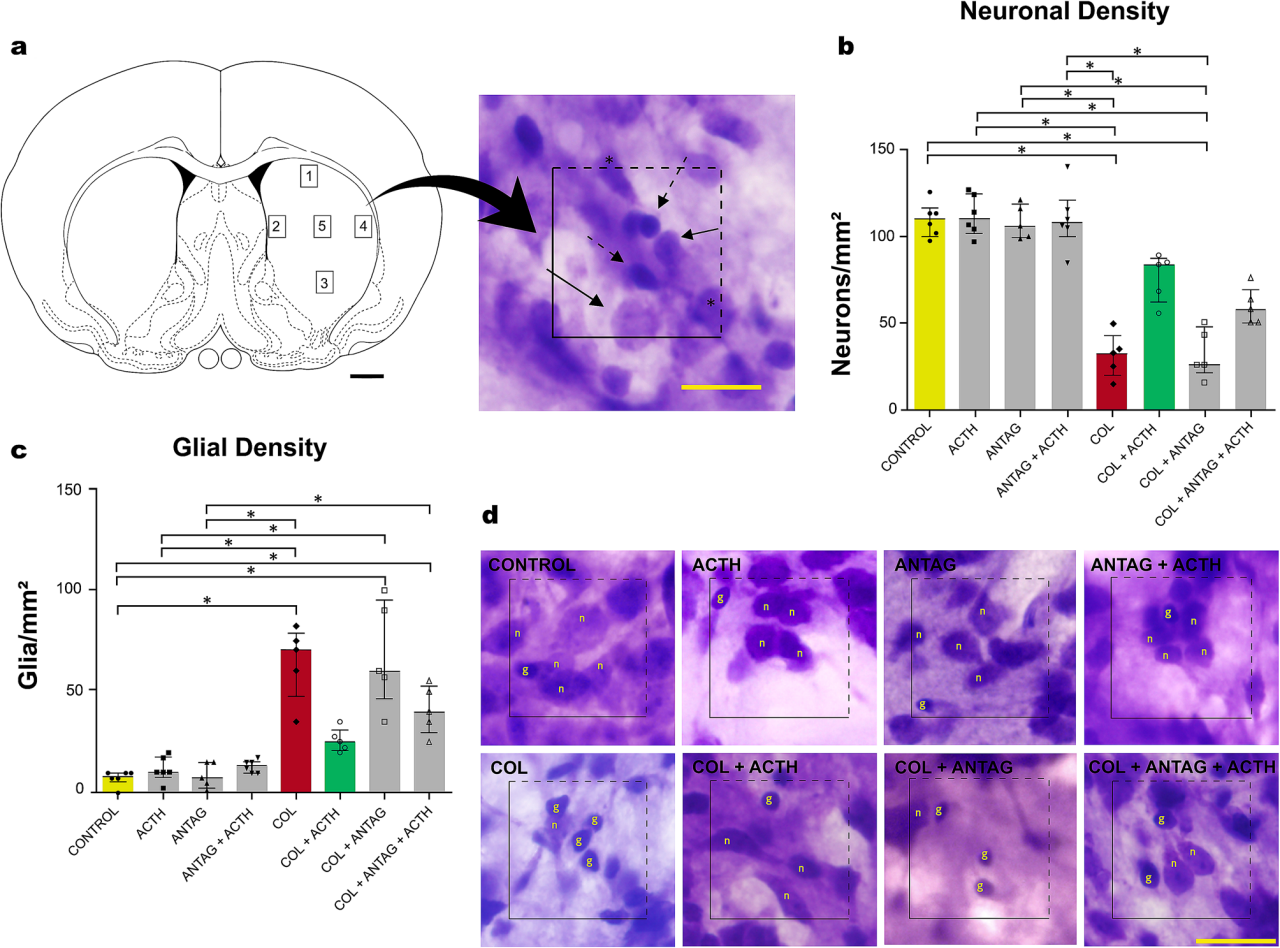
(**a**, left). Stereological procedure for counting cells in the right striatum adjacent to the microinjection site in the germinal matrix. Numbers represent each sampled area. Adapted from^53^. Scale bar = 1 mm. (right) The area of interest (4000 µm^2^) with including (solid lines) and excluding (dashed lines, *not counted cells) borders. Neurons are indicated by thin arrows and glial cells by dashed arrow. Scale bar = 20 µm. (**b**,**c**) Density (median and interquartile ranges) of neurons and glial cells in the right striatum of newborn male rats (n = 5 in “SHAM”, “ANTAG”, “COL”, “COL + ACTH”, “COL + ANTAG” and “COL + ACTH + ANTAG” groups; n = 6 in “ACTH” and “ACTH + ANTAG” groups). Symbols represent values obtained for each rat. *p < 0.05 in each comparison indicated. Note the control values and the effects of collagenase and collagenase plus ACTH. (**d**) Representative images for the number of neurons (n) and glia cells (g) in each experimental group. Scale bar = 20 µm.

**Figure 3:**
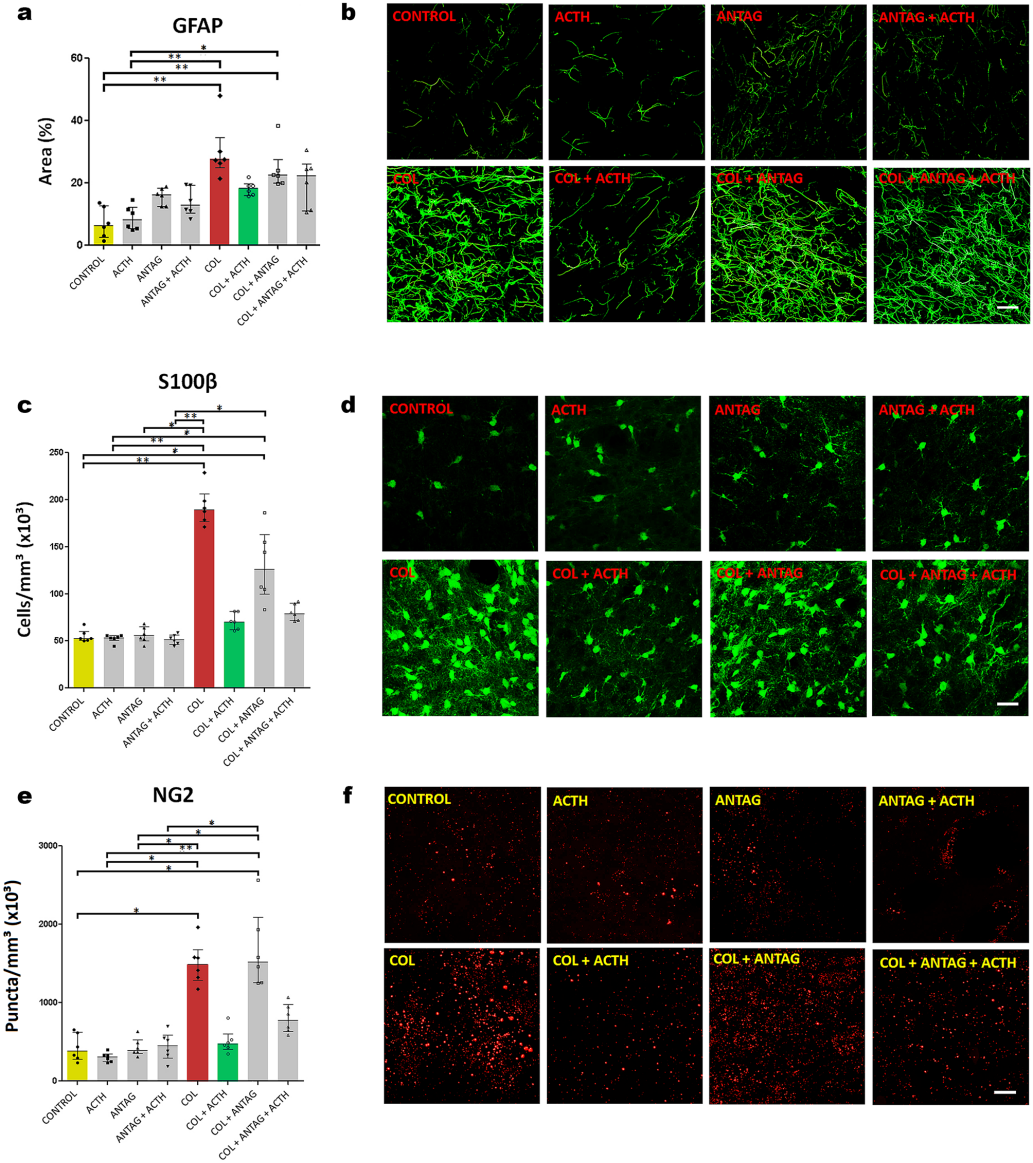
Data (median and interquartile ranges) for (**a**) the area covered by GFAP fluorescent immunoreactivity and the numerical density of (**c**) S100β cells and (**e**) NG2-glia immunoreactive puncta (n = 6 in all groups) in the right striatum adjacent to the microinjection site in the germinal matrix of newborn male rats. Symbols represent values obtained for each rat. *p < 0.05 and **p < 0.01 in each comparison indicated. Note the control values and the effects of collagenase and collagenase plus ACTH. (**b**,**d**, and **f**) are representative images of the corresponding results in each experimental group and biomarker studied. Scale bar = 25 µm.

The PIVH was confirmed using the histological assessment previously described in^12^. Neonatal rats infused with collagenase presented distension of the lateral ventricle, hemorrhage into both the ventricles, and ipsilateral periventricular striatal parenchyma (Fig. 1a; Supplementary Files 1 and 2). The single dose of ACTH_1-24_ 12 h after the collagenase-induced lesion promoted a significant reduction in neuronal and glial losses as well as reduction in the expression of reactive biomarker. For the statistically significant findings described below, the statistical power analysis were: striatal area (right hemisphere, 92.7%), neuronal density (100%), glial density (99.9%), immunoreactive expressions of GFAP (97.2%), S100β (100%), and NG2 (99.9%). The values for the effect size ‘f’ were: striatal area (right hemisphere, 0.75); neuronal density (1.91), glial density (1.28), immunoreactive expressions of GFAP (0.78), S100β (1.58), and NG2 (0.98). Compared to the general control group, main findings for collagenase and the effects of ACTH are highlighted in colors in Figs. 1, 2 and 3.

### PIVH and lesion area

Data are shown in Fig. 1. The microinjections reached the periventricular region, centered in the GM, including the adjacent subependymal region and the striatum in the right hemisphere (Fig. 1a). The induced lesions are related to the experimental procedure in each group. That is, lesions ranged from none to minimal (inherent to the microinjection procedure itself) in all rats of the Control group to intermediary to maximal lesion extensions in the animals composing the COL and the COL + ANTAG groups (Fig. 1a,b). The histological analysis showed that most animals in the group microinjected with ACTH had none to minimal lesions. No animal in the COL + ANTAG + ACTH group showed maximal lesions. The number of rats and the classification of lesion extension in each experimental group are shown in Fig. 1b. There was no mortality of neonates in the present study.

There was an overall statistically significant difference between groups in the lesion extension in the right striatum following microinjections [ANOVA test; F(7,43) = 4.93, *p* < 0.001]. Compared to the Control group, the post hoc comparisons showed that both the lesioned COL and COL + ANTAG groups present a reduced striatal area (Tukey test, *p* < 0.05 in both cases). On the other hand, the striatal area in the COL + ACTH and COL + ANTAG + ACTH groups did not differ from the Control data (Tukey test, *p* > 0.05 in both cases). The Control and ACTH groups showed similar results, i.e., the microinjection of ACTH per se did not alter the striatal area and the results of this group were also higher than the COL and COL + ANTAG ones (Tukey test, *p* < 0.05 in both cases). No other significant difference was found when comparing the other experimental groups (Tukey test, *p* > 0.05; Fig. 1c).

There were no statistically significant differences in the striatal area of the left striatum, contralateral to the microinjection site, between these tested groups [ANOVA test; F(7,43) = 0.94, *p* = 0.48; Fig. 1d).

### Neuronal and glial densities

Data are shown in Fig. 2. There were statistically significant differences between groups in both neuronal density (KW = 34.35; *p* < 0.001; Fig. 2b) and glial density (KW = 35.79; *p* < 0.001; Fig. 2c) in the ipsilateral perilesional area.

The Control, ACTH, ANTAG, and ANTAG + ACTH groups did not differ in the estimated number of neurons in the right striatum. On the other hand, the lowest values were found in the COL and COL + ANTAG groups (Dunn test, *p* < 0.05 in all cases). The ACTH administration led to higher values for the neuronal density in the COL + ACTH and COL + ANTAG + ACTH groups that were not statistically different when compared to the Control data (Dunn test, *p* > 0.05 in both cases). Likewise, the COL + ANTAG + ACTH group did not differ from the Control one, indicating that the effect of ACTH was not fully antagonized by SHU9119 (Fig. 2b,d).

The Control, ACTH, ANTAG, and ANTAG + ACTH groups did not differ in the estimated number of glial cells in the right striatum while the highest values for this parameter were found in the COL and COL + ANTAG (Dunn test, *p* < 0.01 in both cases). The COL group was also different from the ACTH and the ANTAG group, and the COL + ANTAG group was different from the ACTH one (Dunn test, *p* < 0.05 in all cases). The ACTH administration promoted lower values of glial density in the COL + ACTH and COL + ANTAG + ACTH groups, which resemble those found in the Control one (no statistical difference between these groups; Dunn test, *p* > 0.05 in both cases). However, the values of the COL + ANTAG + ACTH group differed from the Control one, indicating that the effect of ACTH was blocked by SHU9119 for this specific parameter (Dunn test, *p* < 0.05; Fig. 2c,d).

### GFAP, S100β and NG2 immunoreactive expression

Figure 3 shows the data and representative images for the biomarkers immunoreactivity in all experimental groups.

There was a statistically significant difference between groups in the area covered by GFAP-immunoreactive cell bodies and processes in the perilesional right striatum (KW = 33.35; *p* < 0.001; Fig. 3a,b). The post hoc comparison showed that Control, ACTH, ANTAG, and ANTAG + ACTH groups have similar results (Dunn test, *p* > 0.05 in all cases). On the other hand, there were higher values in both COL and COL + ANTAG groups than in the Control and ACTH ones (Dunn test, *p* < 0.05 in all cases). These differences were not observed in the COL + ACTH or in the COL + ANTAG + ACTH groups (Dunn test, *p* > 0.05 in all cases; Fig. 3a,b).

There was a statistically significant difference between groups in the density of S100β-immunoreactive cells in the right striatum (KW = 38.76; *p* < 0.001; Fig. 3c,d). The post hoc comparisons showed that the Control, ACTH, ANTAG, and ANTAG + ACTH groups have similar results (Dunn test, *p* > 0.05 in all cases), whereas the COL or the COL + ANTAG groups showed higher values than these former groups (Dunn test, *p* < 0.05 in all cases). These same differences were not found in the groups microinjected with COL + ACTH or COL + ANTAG + ACTH (Dunn test, *p* > 0.05 in all cases; Fig. 3c,d).

Likewise, there was a statistically significant difference between groups in the density of puncta for NG2-immunoreactive glia cells in the right striatum (KW = 36.12; *p* < 0.001; Fig. 3e,f). The post hoc comparisons showed that the Control, ACTH, ANTAG, and ANTAG + ACTH showed similar results (Dunn test, *p* > 0.05 in all cases), whereas the COL or the COL + ANTAG groups showed higher values than these former groups (Dunn test, *p* < 0.05 in all cases). These differences were no longer found in the groups microinjected with COL + ACTH or COL + ANTAG + ACTH (Dunn test, *p* > 0.05 in all cases; Fig. 3e,f).

## Discussion

PIVH is a serious cause of injury to the GM with local neuronal and glial cells losses^12,13,24^. We sought to test the neuroprotective effect of ACTH_1-24_ in a PIVH model, induced by the microinjection of collagenase in the GM at the early postnatal life of male rats. Previous reports studied PN 6 and 7 pups submitted to collagenase-induced PIVH and showed neurological impairments and behavioral deficits^12,13^. Here, morphological and biomarkers data indicate that ACTH_1-24_, 12 h after PIVH injury on PN 2, reduced the periventricular lesioned area, preserving the integrity of local neuronal and glial densities and maintaining low levels of reactive GFAP, S100β and NG2 expressions. SHU9119 blocked the effects of ACTH on glial cells.

The exact route by which ACTH promotes neuroprotection on neurons and glial cells in the neonatal brain is not currently known. Nevertheless, ACTH-related peptides have shown various physiological effects^25^ and neurotrophic/ neuroprotective actions in in vivo and in vitro studies^26^. ACTH_1-24_ acts as a survival and developmental factor increasing neuronal viability in cell culture. Rat embryo cerebral neurons in vitro respond to ACTH_1-24_ displaying a denser neuritic network, which suggests maturation and differentiation of these cells, and show increases in glucose uptake, synthesis of RNA and non-acid-soluble proteins^26^. In chick brain cell cultures, the ACTH_4-9_ analogue ORG 2766 enhances the differentiation of glial cells and induces more GFAP expression [Ref.^26^ and references therein]. ACTH_1-17_ induced significant morphological changes (i.e., rounding of the cell body and process extension) in astroglial cells prepared from primary cultures of 1–2-day-old rat brains^27^. The actions of microglia and immune system cells are reduced by melanocortins^26^. ORG 2766 administered on early postnatal days induces long-lasting neuroprotective mechanisms in brain development, attenuates N-methyl-d-aspartate-induced excitotoxicity, and increases GFAP immunoreactivity in astrocytes in rat nucleus basalis in adulthood^28^. It is also noteworthy that ACTH_1-39_ and α-MSH (acting on MC4) suppressed inflammatory responses, apoptosis, and cellular excitotoxicity in different models of injury in the nervous tissue^19,21–23^. In adrenal cells, signal transduction cascades induced by ACTH involve the arachidonic acid breakdown, adenylate cyclase activation and cAMP synthesis, among other intracellular mechanisms, along with the modulation of cytoskeleton and associated proteins^26,29^. In addition, post-traumatic subcutaneous administration of GMM2, a vasoactive analog of ACTH, effectively reduced disturbances in regional cerebral blood flow such as the early hypoperfusion, blood–brain barrier leakiness, and pathologic elevation of intracranial pressure in a rat model of moderate concussion/brain injury^30^. Since the PIVH pathogenesis is dynamic and complex, other endpoints related to this hemorrhagic injury and ACTH outcome could be tested besides PN 8. Moreover, the evaluation of lesioned brain cytokines, oxidative stress, brain edema and/or other hemosiderin-related neuropathological consequences (in the cerebellar and brain stem structure, for example) are relevant as well^7^. Our above-mentioned findings open new avenues for further experimental efforts to elucidate the mechanism of action and neurotrophic/neuroprotective effects of ACTH_1-24_ in the PIVH rat model.

Reactive gliosis is a hallmark in virtually every type of brain injury, playing a critical role in neural plasticity and recovery. Changes undergone by reactive astrocytes vary with severity of the insult along a graded *continuum* and are regulated in a context-specific manner^31^. PIVH is typically associated with the development of intense reactive astrogliosis^32^. While astrocytes increase the expression of GFAP and S100β in the perilesional region to protect the remaining tissue^31^, they can also undergo intense hypertrophy and proliferation, which may result in anisomorphic astrogliosis and glial scar development^31,33^. Both the astrocytic GFAP gene activation and protein induction participate in astroglial cell activation following a nervous tissue injury. S100β protein regulates astrocyte shape and migration with implications for astrocyte differentiation and activation^34^. NG2-glia engage in rapid signaling with surrounding neurons through direct synaptic contacts, produce new myelin sheaths around injured axons, and contribute to neural repair and recovery^18^. NG2-glia promotes tissue healing, whereas the failure of newly-generated glia to differentiate into functional cells may contribute to reduce the functional efficacy of the repair^18^. Therefore, an effective neuroprotective agent in the acute phase of PIVH should be able to decrease the damage-related reactive astrogliosis as well as be associated with morphological evidence of tissue-saving properties. ACTH_1-24_ was able to reduce injury extension, avoided neuronal and glial losses, and decreased the reactive GFAP, S100β, and NG2 expression in the PIVH-related perilesional area of newborn rats. ACTH_1-24_ per se in the uninjured tissue did not change any of these studied outcomes.

The reduction in such biomarkers expression can indicate a favorable effect of the treatment, also exemplified by few NG2-glia surrounding a damaged area in milder brain injury^35^. It is interesting that resilience responses can modify cell structure and functioning with beneficial or detrimental effects^36^. Astrogliosis can involve adaptive mechanisms for wound repair but, in contrast, can induce harmful effects under specific circumstances^31^. For instance, NG2-glia regulate neuroimmunological mechanisms to maintain cellular proliferation/migration and differentiation into oligodendrocytes, astrocytes or microglia^18,37^. Ablation of NG2-glia can result in increased inflammation, neuronal damage, and atrophy in the hippocampus via activation of an interleukin pathway^37^. We do not have evidence of damaging effects of reduced NG2-immunoreactive puncta on the neuronal or glial density in the present model.

SHU9119 was not able to block all the effects of ACTH. SHU9119 did not reverse the α-MSH anti-inflammatory effects as well^38^, but blocked MCR3/MCR4 actions in regulating feeding behavior^39^. MCR3 and MCR4 can have site-specific and function-specific effects. Furthermore, astrocytes can also express MCR4, whose activation ameliorates reactive phenotype in astrocytes in vitro and increase anti-inflammatory pathways^40^. The doses of SHU9119 used in the current study reduced the protective action of ACTH on the glial cell in the damaged area, but was not able to counteract all the local effects induced by ACTH. It is possible that ACTH-related neuroprotection has also activated other signaling routes. Candidates are the cholinergic anti-inflammatory pathway^41^ or interaction with the dopaminergic neurotransmission^42^. The maintenance and/or restoration of GM intrinsic growth factors would also potentially participate in these preserving effects along the first day of life^6^. Based on the present findings, further studies are necessary to determine the inflammatory process profile as well as the different protein expression patterns after PIVH in the presence of ACTH_1-24_. The use of MCR knock-out animals might provide useful insights for mechanistic studies. In addition, whether blood cells and/or local astrocytes can modulate pro-inflammatory or anti-inflammatory phenotypes following ACTH_1-24_ administration^31^ in PIVH is still a matter of investigation.

Neuroprotective interventions have been tested to reduce PIVH incidence and its severity^7,8^. The prophylactic use of antenatal corticosteroids (e.g., a complete course of betamethasone) reduced the incidence of PIVH in neonates^7,10,43^. However, repeated antenatal glucocorticoid courses can cause adverse effects on fetal growth and cerebral cortical maturation^7^ without further reducing the incidence of PIVH^44^. Postnatal glucocorticoids (dexamethasone or betamethasone) cause a preparation- and dose-dependent reversible reduction in oligodendrocyte proliferation and maturation, reduction in myelination, induced astrogliosis, and impaired motor functions evaluated on postnatal days 14 and 21 in rabbit pups^45^, see also^46^. Here, morphological data indicate that ACTH_1-24_ microinjected 12 h after collagenase in PN 2 alleviated the impact of PIVH in rats. It would be difficult to get permission from parents and the research institution's ethical and regulatory boards to perform an "intracranial injection" treatment test at this moment of ongoing research. However, after considering the benefit-to-harm ratio, intraventricular puncture would provide higher intraparenchymatous levels of ACTH in a short period of time in first PN days. ACTH_1-24_ properties could be further evaluated to determine its suitability for preclinical animal models of PVH^47^, and the possibility to reduce postnatal mortality and sequelae after PIVH injury in premature infants. It is possible that, by actions occurring in a more restricted brain area, ACTH might not induce severe adverse systemic effects, as those related to postnatal steroids actions (e.g., after large dose of dexamethasone) in the early treatment of lung disease of preterm infants^48^. Due to the very small vessels and fragile skin of 1–2 days-old rat pups we have not developed the possibility of systemic administration routes (intravenous or subcutaneous injection) of the ACTH. The drug would also reach its binding sites in the hippocampus, hypothalamus, cerebellum, and brain stem^26^, but systemic infusions would lead to mixed ACTH-related effects (by peripheral binding) as well as undesirable side effects.

In conclusion, our data showed that ACTH_1-24_ reduced the collagenase-induced PIVH lesion in newborn male rats. ACTH_1-24_ also decreased the loss of neurons and glial cells as well as the expression of damage-related biomarkers in perilesional brain tissue. Most of these ACTH effects were not blocked by the use of an antagonist of MCR3/MCR4 and none occurred by ACTH per se in the non-lesioned brain tissue in the present animal model.

## Methods

### Animals

Male Wistar rat pups (N = 48) from the UFCSPA facility were used. The day of birth was considered the PN one. The llitters were standardized to 8 pups per mother. Offspring from different litters composed each experimental group described below. Animals were maintained under standard laboratory conditions, water and food ad libitum, room temperature at 22 °C, and a 12 h-light/dark cycle.

All efforts were made to minimize the number of animals used and their suffering. Rats were manipulated in accordance with national laws and the Council Directive 2010/63EU of the European Parliament and the Council of 22 September 2010 on the protection of animals used for scientific purposes. This study was approved by the local Animal Ethics Committee (UFCSPA, protocol number 506/17).

### PIVH model procedure

Pups were randomly divided into 8 experimental groups (n = 6 in each one). Groups were: (1) “Control”, which received a single microinjection of PBS in the GM; (2) “ACTH”, which received a single microinjection of ACTH_1-24_ (0.048 mg/kg; catalog code A0298, Sigma-Aldrich); (3) “ANTAG”, which received a single microinjection of the MC3/MC4 antagonist SHU9119 (0.01 mg/kg, equivalent to 0.06 nM; catalog code ab141164, Abcam, UK); (4) “ANTAG + ACTH”, which received a single microinjection of both ACTH and SHU9119; (5) “COL”, which received a single microinjection of collagenase; (6) “COL + ACTH”, which received the first microinjection containing collagenase and, 12 h later, a second microinjection containing ACTH at the same doses mentioned above; (7) “COL + ANTAG”, which received the first microinjection of collagenase and, 12 h later, the second microinjection containing SHU9119 at the same doses mentioned above; and, (8) “COL + ANTAG + ACTH”, which received the first microinjection of collagenase and, 12 h later, the microinjections of SHU9119 and ACTH.

**Figure 4:**
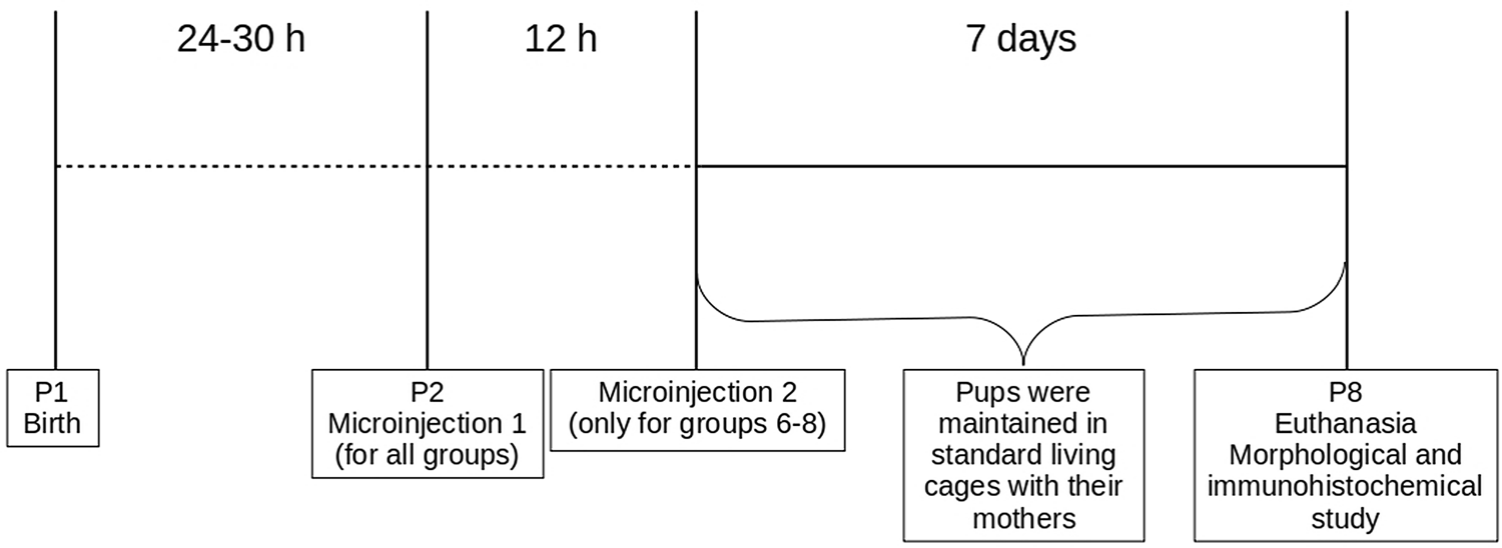
Experimental design and timeline for the induction of peri-intraventricular hemorrhage following the microinjection of collagenase in the germinal matrix of newborn male rats.

The experimental design is shown in Fig. 4. The PIVH was induced 24–30 h after birth, which corresponds to 23–32 weeks of gestational age in humans^49^. We microinjected 0.3 IU of collagenase type VII (catalog number C0773, Sigma-Aldrich, USA) diluted in 2 µl of phosphate buffer solution (PBS; 0.1 M, pH 7.4) directly in the GM. From a pilot study (data not shown), this specific dose of collagenase was effective to spread within the GM and cause hemorrhage in neonatal rats (Supplementary Figs. 1 and 2). This can mimic some aspects of the human brain injury and its neurological consequences^12^. PBS was the vehicle used in previous experiments^12,50–52^ and is recommended by the collagenase manufacturer (Sigma-Aldrich, USA). PIVH and brain parenchyma injury were classified as minimal, intermediary or maximal, as shown in Fig. 1.

Pups on the postnatal day 2 (PN 2) were anesthetized with isoflurane (2% for the induction and 0.5% for anesthesia maintenance), a 30-gauge needle (outer diameter = 0.312 mm) was attached to a 5 µl Hamilton syringe (Hamilton, USA), and percutaneously introduced into the right periventricular region of each newborn animal using a free hand aseptic technique. The microinjection aimed the GM using a custom-made guide to place the needle 3 mm away from the right ear, tangentially to an imaginary line from the ear to the eye and 4 mm below the skull, adapting stereotaxic coordinates from^53^. The place of microinjection was confirmed to be centered in the GM of newborn rats in a pilot study (data not shown). The microinjected volume (2 µL in all groups) was delivered over a period of 5 min and the needle was maintained in the same position for 2 more minutes to prevent backflow. To ensure the best possible results, the needle gauge, the administered volumes, and the infusion rates were adapted from previous reports using this animal model, without evidence of inducing significant lesions per se in control groups^12,13^. After this procedure, pups were maintained on a heated pad (37 °C) during recovery and before returning to their dams. The time elapsed from removing the pups and returning them to the original cage was around 30 min. All mothers accepted their manipulated pups.

The first group was the control one whereas the following groups served to test the effects of ACTH and the MCR3/MCR4 antagonist per se in the present experimental model*.* Afterward, the effects of collagenase and the potential counteracting effects of ACTH with or without the MCR3/MCR4 antagonist were also tested. Collagenase-induced hemorrhage occurs 10 min after infusion and lasts at least 2 h^54^, inducing ongoing parenchymal cellular death and edema^55^. Collagenase-induced damage in the striatum and ventricular dilation of newborn male rats are shown in Supplemental Images 1 and 2. The doses of ACTH and SHU9119 were selected based on previous reports^56,57^ on the′ most effective neuroprotection or receptor antagonism action, respectively. We only used one dose of each substance to avoid drastically increasing the number of experimental groups.

### Histological and fluorescence immunohistochemical procedures

On PN 8, all animals were deeply anesthetized using isoflurane 5% and perfused through the left cardiac ventricle with heparin (1000 IU) followed by 100 ml of 4% formaldehyde in PBS. The brains were removed and postfixed in the same solution at 4 °C for 24 h. Cryoprotection was ensured by dipping brains in 30% sucrose in PBS at 4 °C along 2 days. Coronal sections (50 µm-thick) were obtained along the rostrocaudal axis using a cryostat (Leica, Germany). Alternate serial sections were stained with hematoxylin–eosin (HE) or processed for fluorescent immunohistochemistry. Sequential sections were used for the GFAP, S100β or NG2 study.

The brains of all experimental groups were submitted to the same histological procedures. For the HE technique, brain sections were: (1) stained in hematoxylin solution; (2) washed in tap water; (3) placed in light ammonia solution; (4) washed in tap water; (5) placed in 80% ethanol; (6) counterstained in eosin solution; (7) dehydrated and cleared using ethanol and xylene; and, (8) mounted with synthetic balsam and coverslips. Data were obtained from n = 6 rats in each group, except for the “ANTAG”, “COL + ACTH”, “COL + ANTAG”, and “COL + ANTAG + ACTH” where n = 5 rats.

For the immunohistochemical procedure, brain sections were immersed in 10% methanol and 3% H_2_O_2_ for 30 min, and rinsed in distilled water. The first sections in the sequence were incubated with an anti-GFAP monoclonal antibody conjugated with Alexa Fluor 488 (code 53-9892-82, Invitrogen, USA) diluted 1:500 in PBS containing 0.3% Triton X-100 and 3% albumin bovine serum for 46 h at 4 °C. After rinsing with PBS, the sections were covered with an anti-fading medium solution (Fluoromount; code F4680, Sigma-Aldrich) and coverslips. The second and the third sections in the sequence were processed, respectively, with the anti-S100β monoclonal antibody conjugated with Alexa Fluor 488 (code ab196442, Abcam) diluted 1:100 overnight or the anti-NG2 polyclonal antibody (code ab129051, Abcam) diluted 1:200. For the NG2 study, the secondary antibody used was a rabbit anti-rabbit IgG conjugated with Alexa Fluor 555 (code ab150086, Abcam) at a concentration of 2 µg/ml in PBS-Tx and room temperature for 1 h. Data for each biomarker were obtained for n = 6 pups in each experimental group.

In order to minimize differences in the staining pattern and in the background levels between rats from each experimental group, brains were fixed and post fixed, processed, and incubated in the same medium for the same time. The primary antibody was omitted and replaced by PBS as a control for non-specific labeling induced by the secondary antibody and its related fluorescence. No fluorescent signal was observed (data not shown). The pattern of antibody immunofluorescent expression is in accordance to the manufacturer´s datasheet.

### Quantification of histological and immunohistochemical data

Histological data were obtained for the area of lesion adjacent to the microinjection site in the right striatum. The referential aspect of the normal striatum was checked in both hemispheres of the Control group. The size of the lesioned area in the striatum was measured in sections comprised between 0.70 mm anterior to the bregma to 0.26 mm posterior to the bregma, adapting parameters from^53^. The lesion extension was determined by its histological aspect along the rostrocaudal axis and was arbitrarily classified as minimal, intermediary or maximal (Fig. 1a). The left striatum served as an internal control for the aspect of the striatum in the hemispheric side contralateral to the microinjection procedure.

The lesioned area was estimated by the point count method^12^ using a grid of equidistant points overlaid on the digitized image of the striatum in each brain section studied. The lesioned area was quantified using the following equation:

$$ {\text{A}} = \sum {\text{p}} \cdot {\text{a/p}} $$

where A = estimated area; ∑p = sum of points overlaying the striatum; a/p = point area (1 mm^2^).

The density (number per mm^2^) of neurons and glial cells was evaluated by planar morphometry. Images from HE stained sections were obtained with an Olympus BX-61 light microscope (400×) coupled to a CCD DP72 camera (Olympus, Japan) and the Image Pro-Plus 7.0 software (Media Cybernetics, USA). From each section, five digitized images were obtained from equidistant areas within the striatum (sampling areas numbered 1 to 5 in Fig. 2a). The area of interest (AOI; 4000 µm^2^) was overlaid on the center of each image. All the cells located inside the AOI or intersected by the lower and/or left edges were counted (edges of inclusion). The cells intersected by the upper and/or right edges were not counted (edges of exclusion; Fig. 2a). Neurons showed large, pale nuclei with an evident nucleolus, whereas glia cells had a relatively small size and dense aspect of the chromatin (Fig. 2a,d).

The numerical density of neurons and glial cells was calculated using the formula:

$$ {\text{Nv}} = \, \left[ {{1}/\left( {{\text{a}}/{\text{f}}} \right)} \right) \cdot \left( {\Sigma {\text{Q}}/\Sigma {\text{P}}} \right)] $$

where Nv = estimated numerical density, a/f = area of the counting frame, ΣQ = sum of cells counted, and ΣP = sum of analyzed counting frames. Mean values were calculated for each rat in each experimental group.

Immunofluorescent images of GFAP, S100β and NG2 immunoreactivity in the right striatum were acquired using a confocal microscope with an apochromatic 63×/1.4 water-immersion objective lens (LeicaTCS SP8, Germany). Spectral detectors were adjusted to capture the 520 nm wave-length to visualize the GFAP and the S100β immunoreactivity. Wave-length of 565 nm was used to visualize the NG2 one. Images stacks were acquired along the z axis at each 0.2 µm step with a resolution of 1024 × 1024 pixels. Higher magnification images of representative GFAP, S-100β, and NG2 immunoreactivity results in the right striatum are shown in Supplementary Fig. 3. GFAP and S-100β fluorescence were observed in cell bodies and their extending processes. The expression of NG2 was observed in all animals as immunoreactive puncta, some with varied shapes likely composing cellular segments, as found in previous report (e.g., Fig. 4 in^58^) and in the manufacturer´s datasheet. Further morphological characterization of these NG2 immunoreactive cells in the striatum after injury is desirable and open a new research line^59,60^. Sharpness, color saturation, brightness, and contrast were adjusted using Photoshop CS3 program (Adobe Systems, USA) using the same parameters for all experimental groups.

Relevant expression of these three biomarkers was observed from 2.04 mm anterior to the bregma to 0.12 mm posterior to the bregma^53^. Four equidistant sections were sampled along the rostrocaudal axis per rat in each experimental group. Six AOI were placed over the perilesional area, i.e., 3 AOI in the positions numbered 2 and 5 according to Fig. 2a.

The area covered by GFAP immunolabeled cells was quantified by the binarization technique. This is a reliable and unbiased method comparable to the counting point method to estimate the area covered by GFAP positive cells (Pearson correlation test, *p* = 0.9; data not shown). From each AOI (34,500 µm^2^), we first counted the total number of immunofluorescent pixels and their intensities in each image. It was established the threshold intensity that correlated to the image of a GFAP immunolabeled cell. We used the ImageJ 1.50i software (NIH, USA). Pixel values below this critical value were considered as “background”. Pixel values equal or above it were counted as an immunolabeled point and served to determine the percentage of the sampled area covered by GFAP-positive elements. The tangled aspect of the GFAP processes precluded the identification of isolated astrocytes and further studies of branching radial distribution using the Sholl method^17^.

The stereological estimation of the number of immunomarked S100β and the NG2-glia immunoreactive puncta was done using an adapted optical fractionator approach^17^ and the Image Pro-Plus 6.0 software (Media Cybernetics, USA). The AOI corresponded to 900 µm^2^ for S100β and 100 µm^2^ for NG2-glia data. Counting of the immunolabeled S100β cells and NG2-glia immunoreactive puncta was performed using the including and excluding borders as mentioned above for the density of neurons and glia cells.

The numerical density of immunofluorescent marked S100β cells and NG2-glia immunoreactive puncta was estimated using the following formula:

$$ {\text{Nv}} = \, \left( {{1}/\left[ {{\text{a}}/{\text{f}} \cdot {\text{h}}} \right]} \right) \cdot \left( {\sum {\text{Q}}/\sum {\text{P}}} \right) $$

where Nv = estimated numerical density, a/f = area of the AOI; h = disector height; ∑Q = sum of cells or puncta counted, and ∑P = sum of all analyzed AOI. For each biomarker, mean values per rat in each experimental group were calculated prior to submitting to the statistical tests.

In all cases, data were obtained by a trained researcher blind to the source of the images and experimental groups under study.

### Statistical analysis

Data were tested for normality and homogeneity of variance required for parametric comparisons using the Kolmogorov–Smirnov test and the Bartlett test, respectively. Afterward, the area of lesion was compared between groups using a one-way analysis of variance (ANOVA) followed by the Tukey test for multiple comparisons.

Data corresponding to the number of neurons and glial cells per mm^2^, the area covered by GFAP immunolabeled cells, and the number of S100β cells and NG2-glia immunoreactive puncta per mm^3^ were compared between groups using the Kruskal–Wallis (KW) test followed by the Dunn test for multiple comparisons. Exact values for “*p*” are reported whenever they were available from statistical tables of critical “q” values. The statistically significant level was set a priori at *p* < 0.05.

We used the G*Power 3 software (Institut Für Experimentelle Psychologie, Heinrich Heine Universitat, Düsseldorf, Germany) to calculate the statistical power analysis and the effect size ‘f’, and GraphPad Instat or Prism (version 3.1; GraphPad Software, Inc., USA) for the statistical analyses.

## Supplementary information

Supplementary Figure 1.

Supplementary Figure 2.

Supplementary Figure 3.

Supplementary Legends.

## Data Availability

This study is available at figshare.com using https://doi.org/10.6084/m9.figshare.9729473 and is part of an original M.Sc. thesis (CAM) presented to the Graduate Program in Biosciences (UFCSPA, Brazil) on May 20, 2019.
