# Supplementary figures and images for: Neuroprotective effect of ACTH on collagenase-induced peri-intraventricular hemorrhage in newborn male rats
Source: Sci Rep. 2020 Oct 20;10:17734. doi: 10.1038/s41598-020-74712-7 (PMC7576182; doi:10.1038/s41598-020-74712-7)

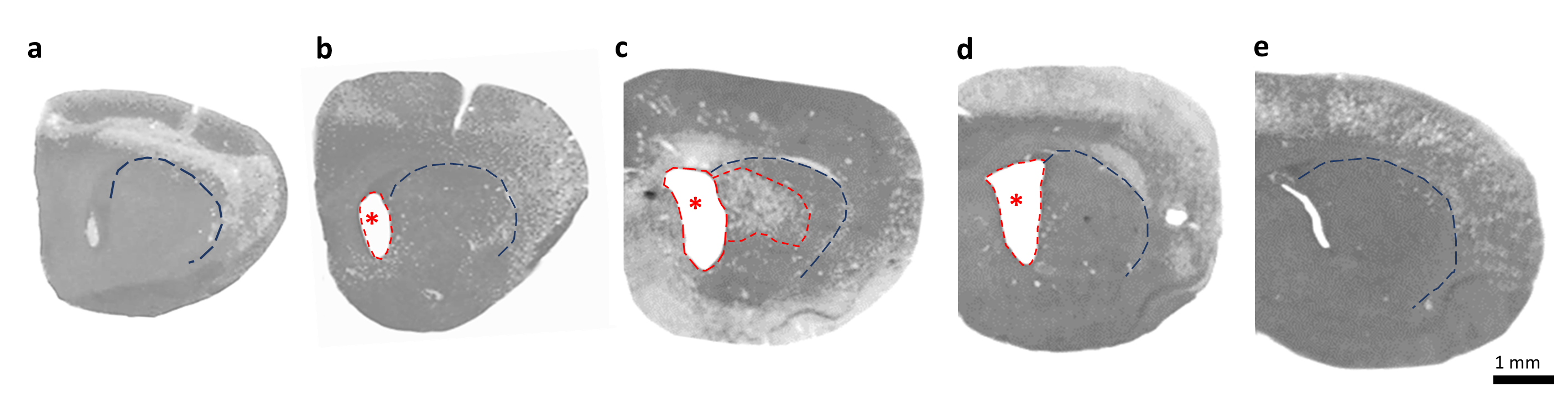

Supplement: Supplementary file 1 — Supplementary Figure 1. [file 41598_2020_74712_MOESM1_ESM.tif]

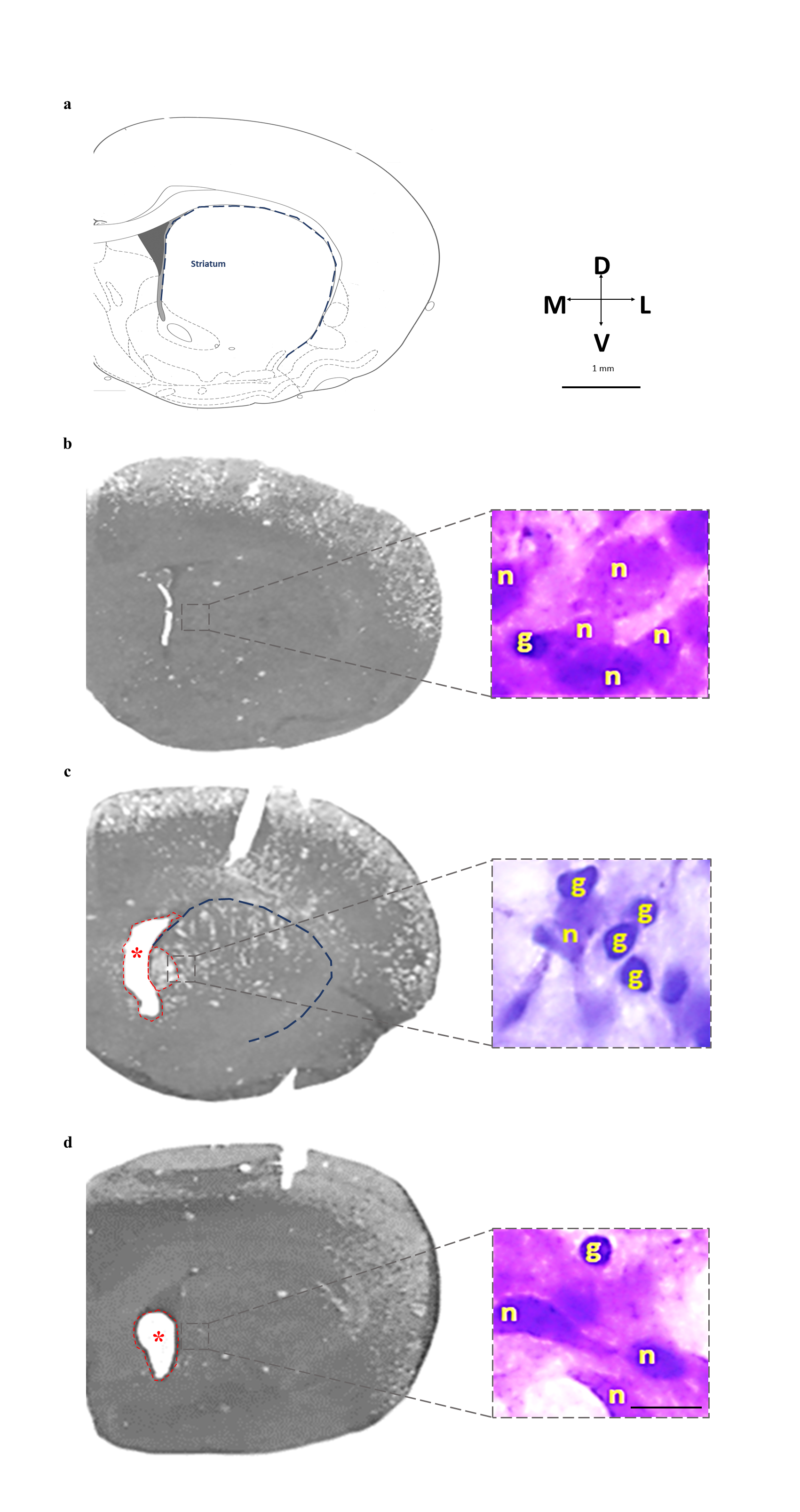

Supplement: Supplementary file 2 — Supplementary Figure 2. [file 41598_2020_74712_MOESM2_ESM.tif]

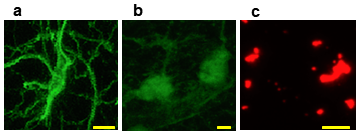

Supplement: Supplementary file 3 — Supplementary Figure 3. [file 41598_2020_74712_MOESM3_ESM.tif]
