# Supplementary material for: Neuroprotective effect of ACTH on collagenase-induced peri-intraventricular hemorrhage in newborn male rats
Source: Sci Rep. 2020 Oct 20;10:17734. doi: 10.1038/s41598-020-74712-7 (PMC7576182; doi:10.1038/s41598-020-74712-7)
Supplement: Supplementary file 4 — Supplementary Legends. [file 41598_2020_74712_MOESM4_ESM.docx]

**Original Research**

**NEUROPROTECTIVE EFFECT OF ACTH ON COLLAGENASE-INDUCED PERI-INTRAVENTRICULAR HEMORRHAGE IN NEWBORN MALE RATS**

**Camila A. Martins^1,2^, Laura Tartari Neves^3^, Marina M.B.P. de Oliveira^3^, Pamela Brambilla Bagatini^3^, Rafaela Barboza^3^, Régis Gemerasca Mestriner^3*^, Léder Leal Xavier^3*^, Alberto A. Rasia-Filho^1,2,4^*^#^**

^1^ Universidade Federal de Ciências da Saúde de Porto Alegre (UFCSPA), Programa de Pós-Graduação em Biociências, Porto Alegre, 90170-050, RS, Brasil.

² Universidade Federal de Ciências da Saúde de Porto Alegre, Departamento de Ciências Básicas da Saúde/Fisiologia, Porto Alegre, 90170-050, RS, Brasil.

^3^ Pontifícia Universidade Católica do Rio Grande do Sul, PUCRS, Escola de Ciências, Laboratório de Biologia Celular e Tecidual, Porto Alegre, 90619-900, Brasil.

^4^ Universidade Federal do Rio Grande do Sul, Programa de Pós-Graduação em Neurociências, Porto Alegre, 90170-050, RS, Brasil.

* These authors contributed equally to the present work.

**Running title:** Neuroprotective effect of ACTH on PIVH

**Number of pages**: 27

**Number of Figures**: 4 + 3 (supplementary)

#**Corresponding author**: A. A. Rasia-Filho, UFCSPA/DCBS/Physiology, R. Sarmento Leite 245, Porto Alegre 90170-050-RS, Brazil. Phone: + 55 51 991161643. E-mail: rasiafilho@pq.cnpq.br; [aarf@ufcspa.edu.br](mailto:aarf@ufcspa.edu.br)

**Supplemenatary Figure 1.** Low magnification microscopic images from rostral (**a**) to caudal (**e**) coronal brain sections (approximately from 0.70 mm to 0.12 anterior to the bregma, adapted from [53]) to exemplify the extension of the collagenase-induced peri-intraventricular hemorrhage injury in the newborn male rat. Collagenase was microinjected in the germinal matrix on postnatal day 2 and animals were studied at postnatal day 8. The ventricle dilation is indicated by an asterisk and the ipsilateral striatal damage by dashed red lines. Injury is shown in its highest point in (**c**).

**Supplementary Figure 2.** Schematic and representative low magnification microscopic images (approximately at 0.96 mm anterior to the bregma) on the collagenase-induced lesion of the germinal matrix (GM) and peri-intraventricular hemorrhage (PIVH) model in newborn male rats. (**a**) Schematic drawing of a coronal section showing the striatum (dashed lines) in the right hemisphere of the rat brain. Adapted from [53]. (**b**) Left: coronal section from a control animal, which received a single microinjection of vehicle in the GM. Note the absence of apparent brain injury signs, including the aspect of the adjacent cerebral ventricle. Right: High magnification microscopic image showing neurons (n) and glia cells (g) in the striatum. (**c**) Left: Collagenase-induced PIVH injury. Collagenase was microinjected in the GM on postnatal day 2 and rats were studied on postnatal day 8. The lateral ventricle dilation is indicated by an asterisk. Right: The ipsilateral striatal injury is evidenced by a reduction in the density of neurons and a high number of glial cells adjacent to the initial hemorrhagic site. (**d**) Left: ACTH_1-24_ microinjected after the collagenase-induced PIVH reduced the area of brain damage and preserved neuronal and glial cell number in the perilesional area. The ventricular dilation is not the same as observed following collagenase alone, and (Right) the density of striatal neurons and glial cells resembles that found in the control group.

**Supplementary Figure 3.** Representative high magnification images (confocal microscopy) showing the fluorescent immunoreactive expression for GFAP (**a**), S100β (**b**), and NG2-glia (**c**) in the striatum of newborn male rats after injury of the germinal matrix using the collagenase-induced peri-intraventricular hemorrhage model. Scale bar = 5 µm.
